# Insulin Tolerance Test in Adult GH Deficiency: An Exploratory Study Deriving BMI-Dependent Cut-Offs Using a Clinical Reference Standard

**DOI:** 10.3390/biomedicines14092001

**Published:** 2026-09-05

**Authors:** Daniela Cuboni, Francesca Mocellini, Michela Sibilla, Giada De Lauro, Emanuele Varaldo, Alessandro Maria Berton, Nunzia Prencipe, Ezio Ghigo, Silvia Grottoli, Mauro Maccario, Valentina Gasco

**Affiliations:** Department of Medical Sciences, Division of Endocrinology, Diabetes and Metabolism, University of Turin, 10126 Turin, Italy; daniela.cuboni@unito.it (D.C.);

**Keywords:** growth hormone deficiency, insulin tolerance test, cut-offs, diagnosis, gold standard, obesity

## Abstract

**Background**: The diagnosis of adult growth hormone (GH) deficiency (GHD) relies on demonstrating a reduced GH response to stimulation tests. Excess body weight, an increasingly prevalent condition, is associated with a blunted GH response to all types of stimulation tests. Therefore, establishing body mass index (BMI)-dependent cut-offs is essential for accurate interpretation. This study aimed to identify BMI-specific diagnostic thresholds for the insulin tolerance test (ITT), using residual pituitary function as the clinical gold standard. **Methods**: We retrospectively analyzed 105 patients with hypothalamic-pituitary disorders who underwent ITT. GHD was defined by the presence of at least three pituitary hormone deficiencies, while preserved somatotropic function was defined by the absence of other pituitary deficits and an insulin-like growth factor-I (IGF-I) standard deviation score ≥ 0. Receiver operating characteristic (ROC) curve analysis was used to determine optimal BMI-stratified cut-offs, defined as those maximizing sensitivity (SE) and specificity (SP). **Results**: The optimal GH cut-off was 2.8 μg/L for patients with normal weight (SE 84.6%, SP 97.4%) and those with overweight (SE 100%, SP 92.3%), and 2.1 μg/L for patients with obesity (SE 88.2%, SP 87.5%). The area under the ROC curve was 0.968, 0.957, and 0.897 for patients with normal weight, overweight, and obesity, respectively. **Conclusions**: This is the first study to define GH diagnostic thresholds for the ITT according to BMI, using a clinical definition of GHD as reference. More restrictive cut-offs in patients with obesity are needed to avoid GHD overdiagnosis and potential overtreatment. These findings should be considered hypothesis-generating and require prospective external validation before routine clinical implementation.

## 1. Introduction

Growth hormone (GH) plays a crucial role not only during childhood and adolescence but also in adulthood, contributing to the maintenance of balanced body composition, healthy metabolism, and optimal physical function. Adult GH deficiency (GHD) has been widely associated with pathological conditions such as increased visceral fat, reduced muscle mass, impaired bone mineral density, and metabolic alterations, including dyslipidemia and insulin resistance [1]. These disturbances not only significantly impact quality of life but also contribute to increased cardiovascular risk and long-term mortality [2].

Numerous studies have shown that replacement therapy with recombinant human GH (rhGH) can significantly improve these conditions, highlighting the crucial role of therapy in restoring metabolic and functional balance in patients with GHD [2,3]. However, despite the evidence of improvements in metabolic and cardiovascular parameters, solid literature on the actual ability of rhGH therapy to normalize long-term mortality remains lacking [4].

A critical issue frequently encountered in the literature on GHD is the variability in its definition, which may contribute to the inconsistent evidence regarding its association with cardiovascular-related mortality [5,6]. Considerable heterogeneity arises from differences in diagnostic tests, GH cut-off values, and assay methodologies. Moreover, patient-related factors—such as the presence of panhypopituitarism, age, and body mass index (BMI)—further complicate data comparability across studies [6]. These issues underscore the need for greater standardization of diagnostic criteria, with particular attention to variables that can influence somatotropic secretion, to better identify patients who may truly benefit from replacement therapy.

Among these variables, BMI is one of the most influential factors affecting GH secretion. The somatotropic response to any stimulus is negatively correlated with BMI [7]. Therefore, GH response cut-off values for GHD diagnosis should be stratified based on BMI categories to avoid false-positive results in overweight or obese patients and false-negative results in normal-weight individuals.

Currently, well-recognized BMI-dependent response cut-offs exist for the Growth Hormone Releasing Hormone + Arginine (GHRH + ARG) test [8], the GHRH + Growth Hormone Releasing Peptide-6 test [9] and the glucagon stimulation test [10]. In contrast, although studies have shown a reduced GH response with increasing BMI in the insulin tolerance test (ITT) [11] and the 2011 guidelines [12] recommend a critical interpretation of the test based on weight category, to date, only one study has attempted to identify BMI-dependent GH cut-off values during the ITT, using the GHRH + ARG test as the gold standard for diagnosing GHD [13]. However, when diagnostic cut-offs are derived using another GH stimulation test as the reference standard, the resulting thresholds may reflect not only the performance of the index test but also the diagnostic limitations and potential misclassification of the reference test itself [14,15].

Based on these premises, the primary objective of this study is to identify BMI-dependent ITT cut-offs for diagnosing adult GHD. Specifically, the study aims to determine these cut-offs in a population consisting exclusively of patients with pituitary disease. This approach improves the robustness of the analysis by reducing the risk of overestimating diagnostic cut-offs that may arise from comparisons with healthy subjects. Furthermore, to minimize errors associated with other available GHD diagnostic tests, case and control group definitions were based solely on clinical criteria.

## 2. Materials and Methods

### 2.1. Study Design and Cohort Definition

A retrospective analysis was conducted on data from patients with hypothalamic-pituitary disorders who underwent an ITT at the Endocrinology, Diabetes and Metabolism Unit of the “Città della Salute e della Scienza” Hospital between 1 January 2021 and 30 September 2024. Data were collected from ITTs performed according to a standardized protocol. At our institution, whenever no contraindications are present, the ITT is routinely used as the reference dynamic test for the evaluation of both somatotropic and corticotropic pituitary function in patients with hypothalamic–pituitary disease. Specifically, the procedure involved the intravenous administration of regular human insulin at a standard dose of 0.10 U/kg for patients without diabetes with BMI < 30 kg/m^2^ and 0.15 U/kg for patients with diabetes or BMI ≥ 30 kg/m^2^. The test was conducted in the morning, starting between 8:00 and 9:00 AM, after an 8–10 h fast, with dual venous access placed beforehand. At baseline (time 0 min), capillary and venous blood glucose, GH, adrenocorticotropic hormone (ACTH) and cortisol levels were measured. Following insulin administration, serial venous blood samples were collected at +30, +45, +60, and +90 min to assess GH, glucose, ACTH and cortisol levels, along with capillary glucose monitoring and documentation of patient-reported hypoglycemic symptoms. The test was considered diagnostically reliable when venous blood glucose dropped below 40 mg/dL and was accompanied by typical hypoglycemic symptoms. In addition to ITT GH peak and glucose nadir data, insulin-like growth factor-I (IGF-I) levels, gender, and age at the time of testing, BMI and pituitary function were recorded. The IGF-I Standard Deviation Score (SDS) was subsequently calculated. Although ACTH and cortisol levels were assessed as part of the standard ITT protocol, given the specific aim of the present study, only data related to the somatotropic axis were included in the analyses and are reported herein.

All patients provided informed consent for data processing. The study was conducted in accordance with the principles of the Declaration of Helsinki and was approved by the Local Ethics Committee “Comitato Etico Territoriale (CET) Interaziendale AOU Città della Salute e della Scienza di Torino” on 25 March 2024 (approval code 0040828).

#### 2.1.1. Definition of the Case–Control Population Based on Clinical Criteria and Derivation of BMI-Dependent Cut-Offs

Additional pituitary hormone deficiencies were diagnosed according to current clinical practice guidelines. Central hypothyroidism and hypogonadotropic hypogonadism were assessed according to the criteria proposed by Fleseriu et al. [16]. For the evaluation of the hypothalamic–pituitary–adrenal axis, morning serum cortisol concentrations < 30 µg/L were considered indicative of central adrenal insufficiency, whereas concentrations > 150 µg/L were considered sufficient. In patients with morning cortisol concentrations between 30 and 150 µg/L, dynamic testing was performed. When not contraindicated, the ITT was used to assess adrenal reserve, with a peak serum cortisol ≥ 181 µg/L considered indicative of an adequate response [16].

The following clinical criteria were adopted as the reference standard for diagnosing or ruling out GHD: according to the Endocrine Society guidelines, GHD was defined by the presence of at least three additional pituitary hormone deficiencies [12]. Conversely, intact somatotropic function was defined as the absence of other pituitary hormone deficiencies together with an IGF-I SDS ≥ 0, as recently proposed [17,18]. Patients with pituitary deficiencies underwent ITT while on stable hormone replacement therapy for at least 6 months.

Exclusion criteria included: presence of only one or two pituitary deficiencies (thyrotropic, corticotropic, and/or gonadotropic function), IGF-I SDS < 0 in patients without pituitary deficiencies, failure to reach a venous blood glucose nadir < 40 mg/dL.

#### 2.1.2. Definition of the Entire Cohort for Secondary Analysis

Patients without additional pituitary deficiencies but with IGF-I SDS < 0, as well as patients with 1–2 pituitary deficiencies, were considered to have an uncertain GHD status and were therefore reintroduced into the study cohort for a secondary exploratory analysis.

### 2.2. Analytical Methods

Quantitative GH determination was performed using an automated chemiluminescent immunoassay (CLIA) in a sandwich format on the LIAISON platform (DiaSorin S.p.A., Saluggia, Italy), employing two monoclonal antibodies specific for human GH. According to the manufacturer’s specifications, the assay had an analytical sensitivity ranging from 0.009 to 0.052 µg/L (defined as the minimum detectable concentration distinguishable from zero by two standard deviations) and a functional sensitivity < 0.05 µg/L. Within-run coefficients of variation (CVs) were 4.4%, 2.4%, 1.6%, 2.1%, and 2.0% at mean GH concentrations of 0.41, 1.19, 3.65, 10.90, and 20.53 µg/L, respectively. Between-run CVs ranged from 5.0% to 10.6% across the same GH concentrations.

Quantitative IGF-I determination was performed using an automated CLIA in a sandwich format on the IDS-iSYS platform (Immunodiagnostic Systems, Boldon, UK). The assay included an acidification step to dissociate IGF-I from its binding proteins, followed by neutralization in the presence of excess IGF-II to prevent reassociation, and employed two monoclonal antibodies specific for IGF-I. The assay had a limit of detection of 4.4 µg/L and a limit of quantification of 8.8 µg/L. Within-run CVs were 2.9%, 2.3%, 1.9%, 1.4%, and 2.3% at mean IGF-I concentrations of 21.9, 81.2, 163, 304, and 663 µg/L, respectively. Total CVs were 5.4%, 3.5%, 3.9%, 7.2%, and 5.3% at the corresponding IGF-I concentrations.

IGF-I levels were expressed both as absolute values and as SDS relative to age- and sex-adjusted reference data derived from a cohort of 15,014 healthy subjects from Europe, Canada, and the United States [19]. SDS values were calculated using the assay-specific equations and transformation method provided in the same publication.

All samples from a single individual were analyzed together to ensure consistency.

### 2.3. Statistical Analysis

#### 2.3.1. Descriptive Statistics

Statistical analysis was performed using MedCalc^®^ version 12.7.0.0 (MedCalc Software Ltd., Ostend, Belgium). Continuous variables were expressed as median and interquartile range (IQR) due to non-normal distribution, while categorical data were expressed as percentages. The distribution of continuous variables was assessed using the D’Agostino–Pearson omnibus normality test, which evaluates departures from normality based on both skewness and kurtosis, before selecting the appropriate parametric or non-parametric statistical analyses. Group differences were evaluated using the Mann–Whitney test for non-parametric continuous variables, the Chi-square and Fisher’s exact tests for categorical variables, and the Kruskal–Wallis test followed by post-hoc analysis to identify significant differences between specific groups. Correlations between continuous variables were assessed using Spearman’s rank correlation coefficient.

#### 2.3.2. Receiver Operating Characteristic Curve Analysis to Determine BMI-Dependent GH Cut-Offs for the Insulin Tolerance Test

To determine the optimal GH response cut-off during ITT for diagnosing GHD, a Receiver Operating Characteristic (ROC) curve analysis was performed only in patients defined as cases or controls according to the clinical definition as specified above. The optimal cut-off was identified using the Youden index, defined as the threshold maximizing the sum of sensitivity (SE) and specificity (SP). Diagnostic cut-offs were calculated based on BMI categories: normal-weight (BMI < 25 kg/m^2^), overweight (BMI 25–29.9 kg/m^2^), and obesity (BMI ≥ 30 kg/m^2^). For each identified cut-off, additional diagnostic performance metrics were calculated, including the positive likelihood ratio (LHR+), negative likelihood ratio (LHR−), positive predictive value (PPV), negative predictive value (NPV), and overall diagnostic accuracy (Acc).

The diagnostic performance of the cut-offs identified in this study was compared with previously proposed cut-offs from the literature [1,12,13,20] in terms of SE, SP, PPV, NPV, LHR+, LHR− and Acc.

#### 2.3.3. Additional Exploratory Analysis

To assess the internal coherence and biological plausibility of the BMI-specific cut-offs derived from the selected case–control population, a secondary exploratory analysis was performed in the overall cohort after reintroducing patients with intermediate clinical phenotypes. A multivariable linear regression model was then constructed on the overall population, including these patients, to identify independent predictors of the GH response to the ITT. The following variables were considered as potential predictors of peak GH at the ITT: age, sex, BMI, nadir blood glucose level during the ITT, number of pituitary hormone deficiencies other than presumed GHD, IGF-I SDS levels, prior neurosurgery, and prior radiotherapy.

Within the same exploratory framework, the prevalence of GHD, as defined by the newly derived BMI-specific cut-offs, was evaluated across increasing numbers of concomitant pituitary hormone deficiencies (0–4) to assess the presence of a biological gradient and the clinical plausibility of the identified thresholds.

## 3. Results

### 3.1. Patient Selection and Study Population Characteristics

Between 1 January 2021 and 30 September 2024, a total of 221 patients with a history of hypothalamic-pituitary disorders underwent an ITT at the Endocrinology, Diabetes and Metabolism Unit of the “Città della Salute e della Scienza Hospital”.

**Figure 1:**
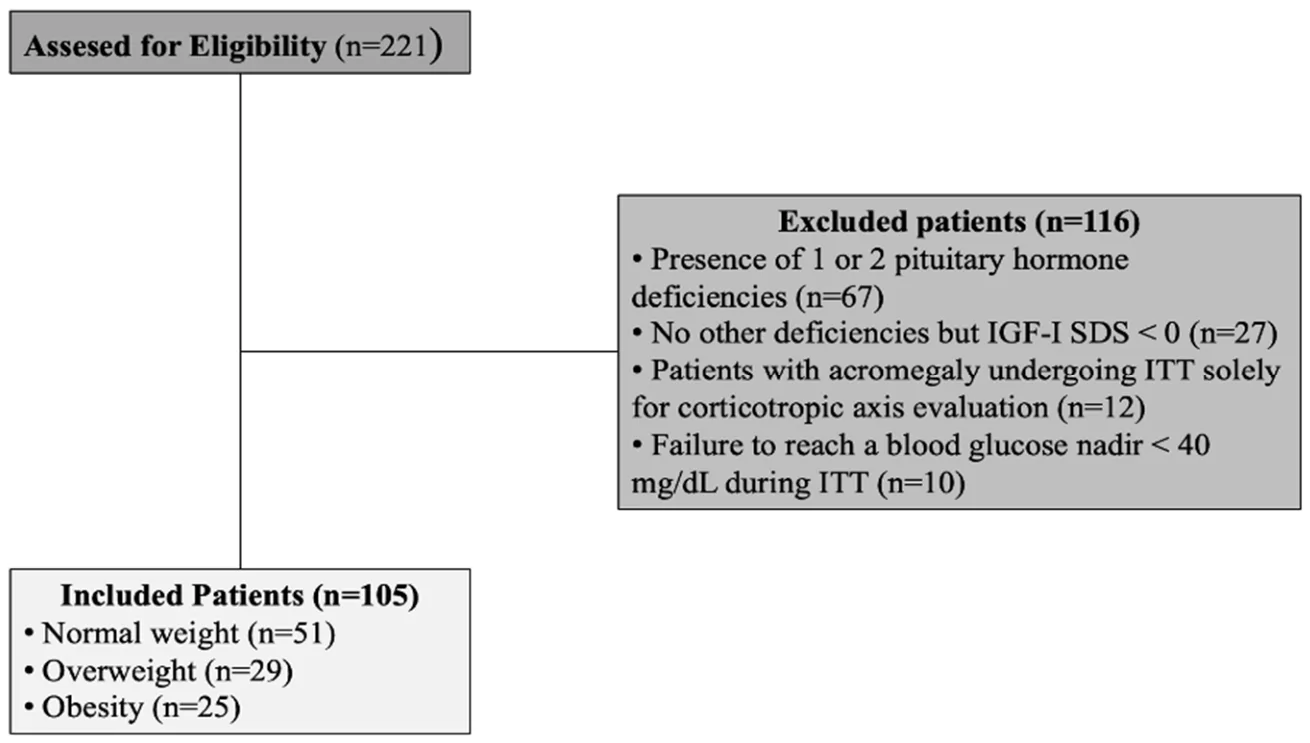
Consort Diagram of the study population.

Figure 1 presents the CONSORT flow diagram of the study population.

The final study population consisted of 105 Caucasian patients (range BMI 17.0–40.3 kg/m^2^), of whom 46 (44%) were diagnosed with GHD (GHD group; range BMI 18.4–40.3 kg/m^2^) and 59 (56%) were classified as not having GHD (non-GHD group; range BMI 17.0–39.1 kg/m^2^), based on the clinical gold standard.

Gender distribution was similar between groups (*p* = 0.293) and did not differ within BMI categories [normal-weight: males GHD vs. non-GHD 69% vs. 58%, *p* = 0.693; overweight: males GHD vs. non-GHD 69% vs. 62%, *p* = 0.989; obesity: males GHD vs. non-GHD 70% vs. 50%, *p* = 0.579]. Patients with GHD were older [41 (32–53) vs. 36 (21–51) years; *p* = 0.041] and had a higher BMI than those without GHD [27.7 (24.4–31.0) vs. 23.4 (21.2–25.9) kg/m^2^; *p* = 0.0003]. However, no significant age differences were observed between GHD and non-GHD groups within BMI subgroups [normal-weight: 35 (29–46) vs. 27 (19–45) years, *p* = 0.230; overweight: 43 (34–49) vs. 50 (38–55) years, *p* = 0.234; obesity: 53 (36–57) vs. 35 (28–51) years, *p* = 0.097], while BMI category distribution differed significantly between groups (*p* = 0.007), with a higher proportion of normal-weight individuals in the non-GHD group (64% vs. 28%; *p* = 0.008). However, the prevalence of overweight (*p* = 0.710) and obesity (*p* = 0.109) did not differ significantly between groups.

The distribution of pituitary dysfunction etiologies differed significantly between groups (*p* = 0.026), with idiopathic isolated childhood-onset GHD more frequent in the non-GHD group (0% vs. 20%, *p* = 0.009).

Overall, 56% of patients had previously undergone neurosurgery (67% GHD vs. 47% non-GHD, *p* = 0.065), and 4% had received radiotherapy (7% vs. 2%, *p* = 0.442).

IGF-I levels were markedly lower in the GHD group [94.5 (60.0–139.0) vs. 219.9 (169.5–317.2) µg/L, *p* < 0.0001], as were IGF-I SDS [−1.20 (−1.75 to −0.66) vs. 0.22 (0.12–0.51), *p* < 0.0001].

A summary of the study population characteristics is provided in Table 1.

### 3.2. GH and Glucose Response to ITT

The peak GH response during ITT was markedly lower in the GHD group compared to non-GHD [0.2 (0.1–1.2) vs. 8.7 (6.0–12.9) µg/L; *p* < 0.0001]. GH peak levels differed across BMI categories (*p* = 0.006), with post-hoc analysis showing higher GH peaks in normal-weight individuals compared to those with obesity (*p* < 0.05). No significant differences were observed between normal-weight and overweight individuals, or between overweight and obese groups.

GH peak did not correlate significantly with the glycemic nadir in the overall population (*p* = 0.219) or within BMI categories (normal-weight: *p* = 0.307; overweight: *p* = 0.760; obesity: *p* = 0.153). Similarly, the glycemic nadir did not differ across BMI categories (*p* = 0.755).

Conversely, glycemic nadir was higher in GHD than non-GHD patients [29.0 (21.0–32.5) vs. 24.0 (18.8–28.0) mg/dL; *p* = 0.047]. However, when stratified by BMI, differences were no longer significant (normal-weight: 29.0 (18.8–35.0) vs. 24.0 (21.0–27.0) mg/dL, *p* = 0.437; overweight: 27.5 (20.5–37.0) vs. 22.0 (17.5–29.3) mg/dL, *p* = 0.128; obesity: 29.0 (24.0–30.0) vs. 23.0 (14.3–32.3) mg/dL, *p* = 0.734).

### 3.3. ROC Curve Analysis

**Figure 2:**
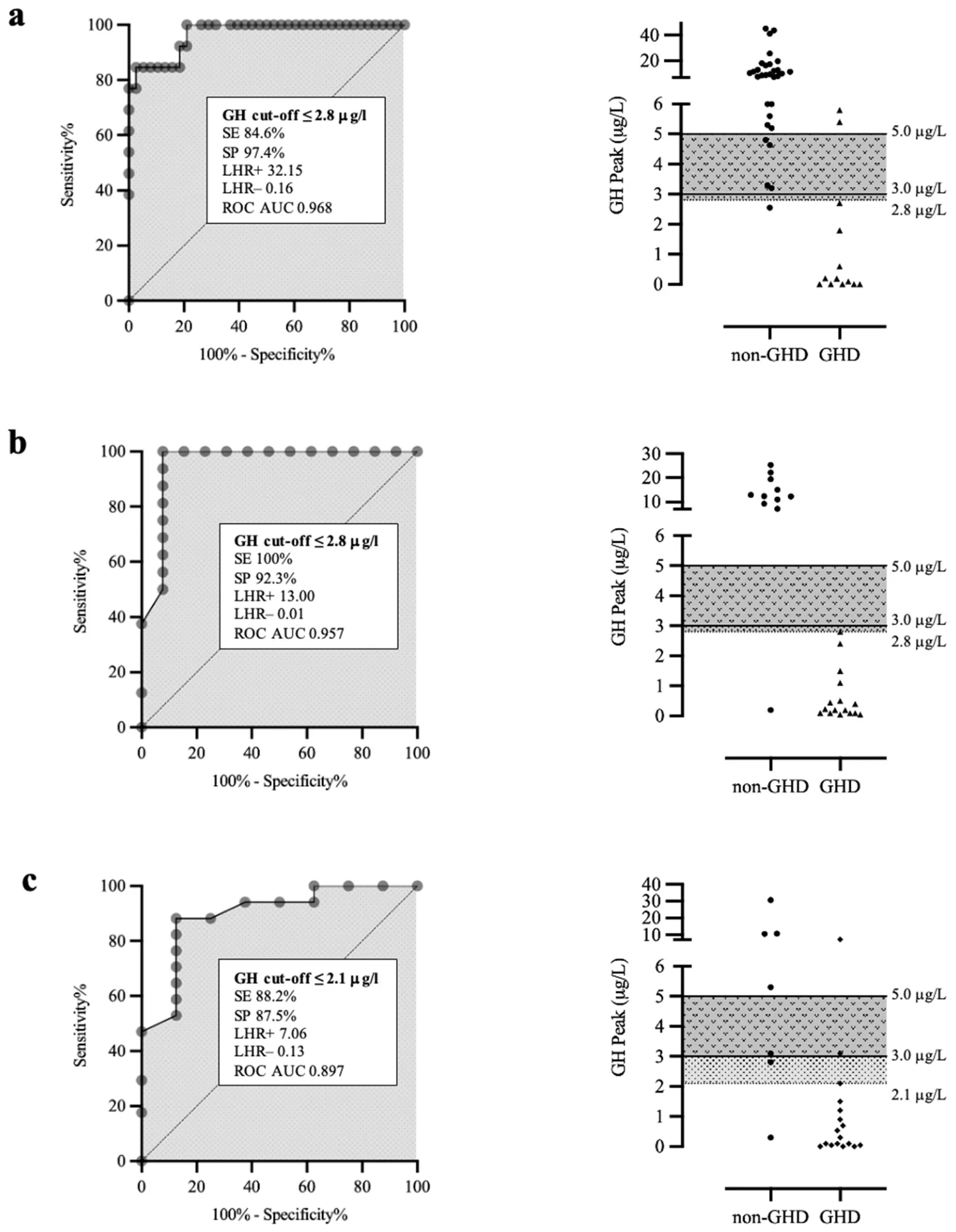
ROC curves for identifying the optimal BMI-specific GH cut-offs during the ITT (**left** panels) and individual GH peak values in patients classified as GHD or non-GHD according to the clinical reference standard (**right** panels). Horizontal lines indicate the BMI-specific cut-offs identified in the present study and the currently recommended guideline thresholds of 3.0 and 5.0 µg/L. Data are shown separately for normal-weight (**a**), overweight (**b**), and obese (**c**) patients.

ROC curve analysis stratified by BMI category identified optimal GH response cut-offs for ITT as follows:

- Normal-weight: 2.8 µg/L (SE 84.6%, SP 97.4%, LHR+ 32.15, LHR− 0.16, PPV 91.7%, NPV 94.9%, Acc 94.1%; area under the curve (AUC) = 0.968) (Figure 2a).
- Overweight: 2.8 µg/L (SE 100%, SP 92.3%, LHR+ 13.00, LHR− 0.01, PPV 94.1%, NPV 100%, Acc 96.6%; AUC = 0.957) (Figure 2b).
- Obesity: 2.1 µg/L (SE 88.2%, SP 87.5%, LHR+ 7.06, LHR− 0.13, PPV 93.8%, NPV 77.8%, Acc 88%; AUC = 0.897) (Figure 2c).

### 3.4. Exploratory Analysis of the Entire Cohort

After reintroducing patients who had been initially excluded due to the presence of one or two additional anterior pituitary hormone deficiencies, or no deficits but IGF-I SDS < 0, a multivariable linear regression analysis was performed on the overall cohort to identify independent predictors of peak GH response to the ITT (Table 2). The final model (n = 100) was statistically significant (*p* < 0.001) and explained 50.9% of the variance in peak GH (R^2^ = 0.509; adjusted R^2^ = 0.466). IGF-I SDS was independently and positively associated with peak GH (β = 2.734, 95% confidence interval (CI) 1.832 to 3.636, *p* < 0.001). BMI was inversely associated with peak GH (β = −0.207, 95% CI −0.357 to −0.057, *p* = 0.007), as was the number of pituitary deficits (β = −1.061, 95% CI −1.741 to −0.381, *p* = 0.003).

**Figure 3:**
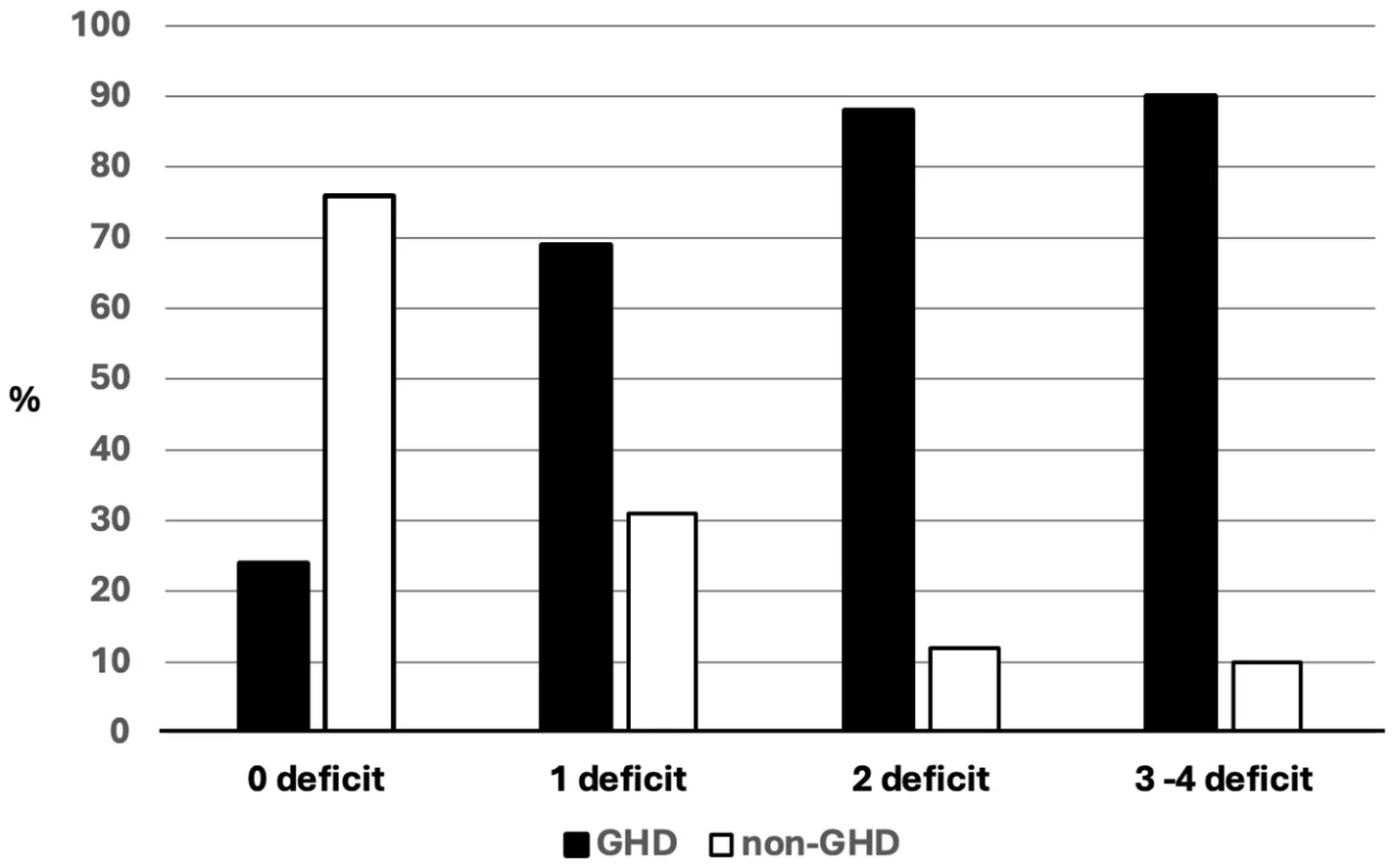
Distribution of subjects classified as growth hormone deficient (GHD) or non-deficient (non-GHD) according to BMI-specific cut-offs across increasing numbers of additional pituitary hormone deficiencies (*p* < 0.0001).

The prevalence of GHD according to the newly derived BMI-specific cut-offs was subsequently assessed across increasing numbers of concomitant anterior pituitary hormone deficiencies (0–4). A significant association was observed between GHD status and the number of additional pituitary deficits (*p* < 0.0001) (Figure 3).

## 4. Discussion

This is the first study to define BMI-specific GH diagnostic cut-offs for GHD using ITT based on a clinical definition of GHD as the gold standard. The proposed cut-offs (≤2.8 µg/L for normal-weight and overweight individuals, and ≤2.1 µg/L for obese patients) support using stricter thresholds in obesity to avoid GHD overdiagnosis and overtreatment. The narrow range of the identified cut-offs (2.1–2.8 µg/L) suggests that, among the stimulation tests used to assess somatotropic function, the ITT may be the least affected by excess body weight.

The clinical presentation of adult GHD is characterized by subtle and non-specific symptoms and signs. Consequently, attributing the clinical picture to somatotropic dysfunction is often particularly challenging. For this reason, only the addition of a history of pituitary damage can guide clinicians towards suspecting GHD. Once GHD is hypothesized, confirmation requires further evaluation, which in most cases includes assessing somatotropic response through stimulation tests [20].

The ITT is considered the gold standard for diagnosing adult GHD by current clinical guidelines, with a diagnostic cut-off defined as a GH peak < 3.0–5.0 μg/L in response to adequate hypoglycemia [1,12,20]. However, despite its high sensitivity, the ITT has several practical and methodological limitations [1,20]. Among these methodological issues, the degree of hypoglycemia required to elicit an adequate GH response remains a matter of debate. A recent pediatric study suggested that a higher glycemic threshold (2.44 mmol/L), in the presence of hypoglycemic symptoms, might preserve diagnostic performance while potentially reducing the risks associated with profound hypoglycemia. However, whether this approach can be safely extrapolated to adults remains to be established [21]. In patients in whom the ITT is contraindicated or considered unsafe, the glucagon stimulation test represents a valuable alternative for the assessment of GH reserve, as it does not require the induction of profound hypoglycemia. However, the test requires prolonged sampling, and its interpretation may be influenced by patient-related factors, including BMI and glycemic status [6]. Moreover, its use in clinical practice remains relatively limited [22,23].

Nonetheless, in a recent audit on the management of adult GHD sponsored by the European Society of Endocrinology (ESE), the ITT, along with the GHRH + ARG test, was found to be the most frequently used diagnostic tool for GHD [22]. Given the current unavailability of GHRH—and the resulting inability to perform the GHRH + ARG test—the ITT may once again become the most widely used diagnostic test for adult GHD. This possibility is further supported by recent alerts regarding the unavailability of glucagon, at least in some countries (https://bit.ly/AIFAdoc (accessed on 28 July 2026); https://bit.ly/CriticalMedicinesSupplyChain (accessed on 28 July 2026)), as well as by the high cost and limited availability of the macimorelin test in the United States [20,24].

At this point, critically analyzing the evidence supporting the ITT as the diagnostic gold standard is essential to identify potential issues limiting its reliability. GH diagnostic cut-offs in response to ITT for GHD diagnosis were initially proposed by Hoffman et al. [14] in 1994 based on GH response to hypoglycemia in 23 patients with organic pituitary disease compared to 35 healthy subjects matched for age and BMI. Beyond the evident issue of small sample size, the study suffered from a critical methodological flaw: comparing GH responses between patients with pituitary disease and healthy subjects, despite matching for age, sex, and BMI, introduces a risk of overestimating diagnostic cut-off accuracy. Furthermore, applicability is complicated by the lack of BMI-dependent cut-offs, despite overweight and obesity being well-established negative regulators of somatotropic response [7,25,26,27]. Moreover, the GH cut-off identified in that study, set at 3.0 µg/L, was based on outdated and non-specific assay methods, which may have led to an overestimation of the identified cut-off. Based on Biller et al. [15], the diagnostic cut-off was later revised, increasing the threshold from 3.0 to 5.0 μg/L. However, this study included obese subjects in the control group, introducing a significant confounding factor and increasing the risk of overdiagnosis in normal-weight individuals.

In summary, the guideline-recommended cut-offs derive from studies with very limited sample sizes, suffer from the critical methodological flaw of comparing GH responses between patients with pituitary disease and healthy individuals, and fail to identify specific cut-offs for normal-weight, overweight, and obese patients.

To overcome these limitations, our group previously attempted to identify BMI-specific ITT cut-offs in 2021, using the GHRH + ARG test as the gold standard for GHD diagnosis [13]. However, despite including a larger cohort of patients with hypothalamic-pituitary disease, that study had a major methodological limitation: the use of another stimulation test to define GHD meant that the identified cut-offs were inherently influenced by the sensitivity and specificity of the test used as the reference standard. Therefore, to overcome the limitations of previous studies, we aimed to identify BMI-related GH response cut-offs for the ITT in a cohort of 105 patients with hypothalamic-pituitary disease. Residual pituitary function was used as the clinical gold standard to define cases and controls, thereby avoiding reliance on another GH stimulation test. Indeed, further investigations are unnecessary to diagnose GHD in patients with at least three additional pituitary hormone deficiencies [12], as previous studies have shown that the probability of GHD in such individuals is as high as 97% [20,28]. Similarly, patients with hypothalamic-pituitary disease without additional deficits (except suspected GHD) and with IGF-I SDS ≥ 0 were considered unlikely to have clinically significant severe GHD [17,18]. We acknowledge that normal IGF-I concentrations alone cannot exclude adult GHD. However, serum IGF-I correlates with the severity of somatotropic dysfunction rather than representing an absolute diagnostic criterion [29]. Consequently, in our study, IGF-I was not used to exclude GHD per se, but rather to improve the specificity of the control group by minimizing the inclusion of subjects with isolated or severe GHD. Conversely, requiring an IGF-I SDS < −2 in patients with multiple pituitary hormone deficiencies would have restricted the GHD group to a small subgroup with the most profound biochemical phenotype, reducing the representativeness of the study population without improving the clinical validity of the adopted gold standard. Analysis of patient characteristics in both groups supports the validity of the chosen clinical gold standard. Non-GHD patients had a lower BMI and a higher prevalence of normal-weight, consistent with the absence of phenotypic features typically associated with GHD [30].

Regarding the etiology of hypothalamic-pituitary damage, differences were mainly due to the higher prevalence of childhood-onset idiopathic deficiency in the non-GHD group. This distribution reflects the inclusion criteria, and the high likelihood of resolution of idiopathic childhood-onset deficiency in adulthood [20], along with normal IGF-I levels, explains why this condition was the second most frequent among controls.

No significant differences were observed between groups in prior neurosurgical or radiotherapy treatments. However, the *p*-value for neurosurgery approached significance, consistent with the well-known association between neurosurgical interventions and increased risk of hypopituitarism and subsequent GHD. A similar rationale applies to radiotherapy, where the lack of differences likely reflects the low number of treated individuals in the cohort.

The difference in etiology between groups likely accounts for the younger age of subjects without GHD. However, this age difference is not expected to compromise study validity. Although GH response to ITT may decline with age [31], this was not observed in our multivariable analysis, and the age difference observed in our sample (41 vs. 36 years in the GHD and non-GHD groups, respectively) is not clinically meaningful. Regarding the possible impact of sex on ITT response [6,32,33], the comparable distribution of males and females in both groups minimizes potential biases in GH peak interpretation. Furthermore, our multivariable analysis showed that sex was not significantly associated with GH response to ITT.

Based on these premises, we identified the following BMI-stratified cut-off values:

- Normal-weight: 2.8 μg/L
- Overweight: 2.8 μg/L
- Obesity: 2.1 μg/L

For normal-weight patients, the identified cut-off closely aligns with the 3.0 μg/L threshold proposed by Hoffman et al. [14]. However, the 1994 study used a radioimmunoassay (RIA), which generally yields higher absolute GH values than the chemiluminescence assay employed in the present study [14]. Therefore, direct comparison is limited, and the 2.8 μg/L cut-off appears more appropriate for contemporary assay techniques.

The ROC curve showed an AUC approaching 1, indicating excellent discriminatory power and optimal diagnostic performance. High specificity (>95%) and good sensitivity (approximately 85%), together with LHR+ > 10, LHR− < 0.2, and PPV and NPV between 90% and 95%, support the utility of this threshold both for confirming GHD in high-risk individuals and for ruling it out in low-risk cases.

In overweight patients, the same cut-off (2.8 μg/L) was identified. Consistently, GH peak values differed significantly only between normal-weight and obese patients, with overweight individuals showing intermediate values. Diagnostic performance in this group was also optimal.

In obese patients, the optimal cut-off (2.1 μg/L) was lower than the 3.0–5.0 μg/L threshold recommended by current guidelines [1,12,20]. The AUC confirmed good test validity, with an overall diagnostic accuracy of 88%. Although slightly lower than in normal-weight and overweight groups, diagnostic performance remained high.

Notably, the three identified cut-offs fell within a narrow range (2.1–2.8 μg/L), suggesting that, among stimulation tests used to assess somatotropic function, the ITT is the least influenced by excess body weight [11]. However, as demonstrated in the secondary analysis, BMI independently emerged as a negative predictor of GH peak during ITT in the multivariable model. This association does not appear to be mediated by a lesser degree of hypoglycemia in individuals with excess body weight—potentially reflecting greater insulin resistance—as glycemic nadir was not independently associated with GH peak. These findings suggest that the negative impact of BMI on GH peak is unlikely to be explained by differences in hypoglycemic response during ITT.

Comparison of the cut-offs identified in this study with those recommended by current guidelines [1,12,20] and with the previously proposed BMI-adjusted thresholds from our group [13] (Table 3) shows that, in normal-weight patients, sensitivity was similar across all thresholds. However, higher cut-offs (3.0–5.0 μg/L) reduced specificity and increased false positives—particularly at 5.0 μg/L—potentially exposing patients to replacement therapy with an unfavorable risk–benefit ratio.

In overweight individuals, the 2.8 μg/L cut-off demonstrated diagnostic performance comparable to the 3.0–5.0 μg/L thresholds recommended by guidelines, whereas the previously proposed 1.3 μg/L threshold [13], despite identical specificity, showed lower sensitivity and overall accuracy due to more false negatives.

In obesity, applying the 3.0–5.0 μg/L guideline cut-offs [1,12,20] increased the risk of overdiagnosis. Notably, the PPV at 5.0 μg/L decreased from 93.8% (observed with the 2.1 μg/L cut-off) to 84.2%, reflecting a higher proportion of false positives.

Overall, if specificity is prioritized to ensure that only truly deficient patients receive rhGH therapy, a cut-off of 2.8–3.0 μg/L in normal-weight and overweight patients and 2.1–2.2 μg/L in those with obesity appears more appropriate.

Importantly, comparisons with historical ITT thresholds should be interpreted in light of the substantial evolution of GH assay methodology over time. Several widely used ITT cut-offs were derived using older GH assays with lower specificity than contemporary immunometric methods, which may limit their direct applicability to current clinical practice. More recent studies using contemporary assays continue to support the diagnostic role of the ITT, although evidence specifically addressing BMI-dependent thresholds remains limited [34,35].

To further assess the robustness of our findings beyond the strict case–control comparison, a secondary exploratory analysis was performed in the overall cohort after reintroducing intermediate phenotypes. Although BMI-specific thresholds were derived from a selected case–control sample to maximize diagnostic contrast, this extended analysis aimed to evaluate their internal coherence and biological plausibility. In the multivariable model, IGF-I SDS, number of pituitary deficiencies, and BMI independently predicted peak GH response to ITT. The independent associations of IGF-I SDS and the number of pituitary deficiencies with GH peak support the coherence of the adopted clinical gold standard, as lower IGF-I SDS and a greater number of deficiencies were consistently linked to a blunted somatotropic response, irrespective of BMI.

Moreover, applying the newly derived BMI-specific cut-offs across increasing numbers of pituitary hormone deficiencies revealed a progressive biological gradient in GHD prevalence, further supporting the clinical plausibility of the proposed thresholds.

Overall, the concordance between multivariable predictors of GH response and the observed biological gradient strengthens the validity of the proposed BMI-specific diagnostic cut-offs. However, these findings were interpreted as an assessment of the internal consistency of the proposed BMI-specific cut-offs rather than as an external validation of their diagnostic performance.

This study has several strengths. Its monocentric design ensured homogeneous patient selection, test execution, and data interpretation, thereby enhancing internal validity. The use of a second-generation chemiluminescent immunoassay provided assay-specific and consistent results. Despite the rarity of adult GHD, the relatively large sample enabled meaningful subgroup analyses, particularly by BMI category. Furthermore, the clinical gold standard based on residual pituitary function minimized the limitations of previous studies relying on dynamic tests. Nevertheless, the findings should be interpreted as exploratory and hypothesis-generating. Although the case–control design allowed the derivation of cut-offs from clinically well-characterized populations, prospective validation in larger and more heterogeneous cohorts, including patients with intermediate clinical phenotypes, will be required before these thresholds can be considered for routine clinical use.

Some limitations should also be acknowledged. First, all participants were of Caucasian ethnicity, which may limit the generalizability of the findings to other ethnic populations. Furthermore, BMI does not provide a direct assessment of body composition and may imperfectly reflect adiposity in patients with GHD, who typically exhibit reduced lean body mass and increased fat mass. Therefore, patients within the same BMI category may have substantially different proportions of fat and lean mass. More accurate body composition assessments, such as dual-energy X-ray absorptometry or bioelectrical impedance analysis, were not systematically available in this retrospective cohort. This limitation should be considered when interpreting the BMI-specific GH cut-offs identified in the present study. Second, GH and IGF-I measurements are subject to inter-assay variability. In particular, the GH cut-off values identified in this study should be considered assay-specific, given the well-recognized lack of standardization across GH immunoassays. Although IGF-I was expressed as SDS, thereby reducing methodological heterogeneity, some residual variability related to assay-specific reference populations cannot be excluded. In addition, gonadal function and sex-steroid replacement therapy were not specifically considered in the present analyses. Future studies should evaluate their potential influence on BMI-specific ITT cut-offs through dedicated stratified analyses. Finally, although isolated GHD cases may theoretically have been included in the control group, the requirement for an IGF-I SDS ≥ 0 likely reduced the risk of including patients with severe isolated GHD, thereby limiting misclassification bias. Conversely, the clinically defined gold standard, while biologically sound, may have preferentially identified well-characterized phenotypes and underrepresented borderline cases.

## 5. Conclusions

Our study proposes clinically derived BMI-dependent GH cut-offs for the ITT that demonstrated high diagnostic performance within a carefully characterized cohort of patients with hypothalamic-pituitary disease, providing a rationale for future prospective validation. The proposed thresholds (2.8 μg/L for normal-weight and overweight patients; 2.1 μg/L for obesity) showed high diagnostic performance, with AUC values approaching 1. The use of a clinically defined gold standard—rather than comparisons with healthy controls or alternative stimulation tests—strengthened the reliability of our findings. Our data indicate that applying generic cut-offs (3.0–5.0 μg/L) may increase the risk of overdiagnosis, particularly in patients with obesity. Incorporating BMI-specific thresholds improves diagnostic stratification and supports more accurate identification of patients most likely to benefit from replacement therapy. Finally, the progressive biological gradient observed across increasing numbers of pituitary hormone deficiencies further reinforces the validity and clinical coherence of the proposed BMI-adjusted cut-offs. Prospective external validation in larger, multicenter cohorts and across different assay platforms will be essential before they can be adopted in routine clinical practice.

## Figures and Tables

**Table 1:** Clinical and biochemical characteristics of the study cohort stratified by growth hormone deficiency defined by clinical criteria.

	All Study Population (n = 105)	GHD (n = 46)	Non-GHD (n = 59)	*p*
**Gender** n. (%)				0.293
Male subjects	66 (63)	32 (70)	34 (58)	
Female subjects	39 (37)	14 (30)	25 (42)	
**Age** years median (IQR)	38 (27–51)	41 (32–53)	36 (21–51)	0.041
**BMI** kg/m^2^ median (IQR)	25.4 (21.5–29.4)	27.7 (24.4–31.0)	23.4 (21.2–25.9)	0.0003
**BMI category** n. (%)				0.007
Normal-weight	51 (48)	13 (28)	38 (64)	0.008
Overweight	29 (28)	16 (35)	13 (22)	0.710
Obesity	25 (24)	17 (37)	8 (14)	0.109
**Pituitary disease** n. (%)				0.026
Pituitary adenoma	57 (54)	27 (59)	30 (51)	0.791
Craniopharyngioma/Rathke’s cleft cyst	10 (10)	7 (15)	3 (5)	0.343
Primary empty Sella	14 (13)	7 (15)	7 (12)	0.789
Idiopathic CO-GHD	12 (11)	0 (0)	12 (20)	0.009
TBI or SAH n.	2 (2)	1 (2)	1 (2)	0.479
Other ^#^	10 (10)	4 (9)	6 (10)	0.752
**Neurosurgery** n. (%)	59 (56)	31 (67)	28 (47)	0.065
**Radiotherapy** n. (%)	4 (4)	3 (7)	1 (2)	0.442
**IGF-I** median (IQR) μg/L	149.0 (89.5–214.3)	94.5 (60.0–139.0)	219.9 (169.5–317.2)	<0.0001
**IGF-I SDS** median (IQR)	0 (−1.27–0.27)	−1.20 (−1.75–−0.66)	0.22 (0.12–0.51)	<0.0001
**GH peak to ITT** μg/L median (IQR)	5.2 (0.3–9.6)	0.2 (0.1–1.2)	8.7 (6.0–12.9)	<0.0001
**Glycemic nadir** mg/dL median (IQR)	25.0 (20.0–30.0)	29.0 (21.0–32.5)	24.0 (18.8–28.0)	0.047

BMI: body mass index; CO-GHD: childhood onset growth hormone deficiency; GHD: growth hormone deficiency; IGF-I: insulin-like growth factor-I; IQR: interquartile range; ITT: insulin tolerance test; normal weight: BMI < 25 kg/m^2^, overweight BMI: 25–29.9 kg/m^2^, obesity: BMI ≥ 30 kg/m^2^; SDS: standard deviation score; SAH: subarachnoid hemorrhage; TBI: traumatic brain injury. ^#^ Other causes category: the GHD group included one patient with histiocytosis, two with idiopathic panhypopituitarism, and one with granulomatous disease. The non-GHD group included two patients with pituitary hyperplasia, two with hypothalamic-pituitary malformations, one with hypophysitis, and one with amyloidosis.

**Table 2:** Multiple linear regression analysis of factors associated with GH peak during the insulin tolerance test. Regression coefficients (β), standard errors (SE), 95% confidence intervals and *p* values are shown for each independent variable included in the model.

Variable	β	SE	95% CI	*p*
BMI	−0.207	0.076	−0.357 to −0.057	0.007
Age at test	−0.050	0.035	−0.119 to 0.020	0.161
IGF-I SDS	2.734	0.454	1.832 to 3.636	<0.001
Number of pituitary hormone deficits	−1.061	0.343	−1.741 to −0.381	0.003
Sex	−0.384	0.848	−2.068 to 1.299	0.651
Glucose nadir	0.022	0.040	−0.058 to 0.102	0.584
Previous radiotherapy	−1.862	2.012	−5.858 to 2.134	0.357
Previous neurosurgery	−0.176	0.984	−2.130 to 1.778	0.858

Model summary: n = 100, R^2^ = 0.509, Adjusted R^2^ = 0.466, F(8,91) = 11.8, *p* < 0.001 BMI, body mass index; CI: Confidence Interval; IGF-I, insulin-like growth factor I; SDS, standard deviation score.

**Table 3:** Diagnostic performance of identified BMI-dependent growth hormone cut-offs compared with literature thresholds.

	Cut-Off (μg/L)	SE (%)	SP (%)	PPV (%)	NPV (%)	LHR+	LHR−	Acc (%)
**Normal weight**								
This study	2.8	84.6	97.4	91.7	94.9	32.15	0.16	94.1
Hoffman et al. 1994 [14]	3.0	84.6	97.4	91.7	94.9	32.15	0.16	94.1
Biller et al. 2002 [15]	5.0	84.6	86.8	68.7	94.3	6.41	0.18	86.2
Gasco et al. 2021 [13]	3.5	84.6	92.1	78.6	94.6	10.71	0.17	90.2
**Overweight**								
This study	2.8	100	92.3	94.1	100	13.00	0.01	96.6
Hoffman et al. 1994 [14]	3.0	100	92.3	94.1	100	13.00	0.01	96.6
Biller et al. 2002 [15]	5.0	100	92.3	94.1	100	13.00	0.01	96.6
Gasco et al. 2021 [13]	1.3	81.2	92.3	92.9	80	10.55	0.20	86.2
**Obesity**								
This study	2.1	88.2	87.5	93.8	77.8	7.06	0.13	88.0
Hoffman et al. 1994 [14]	3.0	88.2	75.0	88.2	75.0	3.53	0.16	84.0
Biller et al. 2002 [15]	5.0	94.1	62.5	84.2	83.3	2.51	0.09	84.0
Gasco et al. 2021 [13]	2.2	88.2	87.5	93.8	77.8	7.06	0.13	88.0

## Data Availability

The dataset generated and analyzed during the current study is available from the corresponding author on request.

## Abbreviations

The following abbreviations are used in this manuscript:

Acc	Accuracy
ACTH	Adrenocorticotropic Hormone
AUC	Area Under the Receiver Operating Characteristic Curve
BMI	Body Mass Index
CI	Confidence Interval
CLIA	Chemiluminescent Immunoassay
CO-GHD	Childhood-Onset Growth Hormone Deficiency
CV	Coefficient of Variation
ESE	European Society of Endocrinology
GH	Growth Hormone
GHD	Growth Hormone Deficiency
GHRH	Growth Hormone-Releasing Hormone
GHRH + ARG	Growth Hormone-Releasing Hormone plus Arginine
GHRP-6	Growth Hormone-Releasing Peptide-6
IGF-I	Insulin-Like Growth Factor-I
IQR	Interquartile Range
ITT	Insulin Tolerance Test
LHR+	Positive Likelihood Ratio
LHR−	Negative Likelihood Ratio
NPV	Negative Predictive Value
PPV	Positive Predictive Value
rhGH	Recombinant Human Growth Hormone
RIA	Radioimmunoassay
ROC	Receiver Operating Characteristic
SAH	Subarachnoid Hemorrhage
SDS	Standard Deviation Score
SE	Sensitivity
SP	Specificity
TBI	Traumatic Brain Injury
